# Venous congestion in critical care: combining VExUS, lung ultrasound, and echocardiography

**DOI:** 10.1016/j.aicoj.2026.100127

**Published:** 2026-07-25

**Authors:** Karel Huard, Jean Deschamps, Etienne J. Couture, Marc-Antoine Lepage, William Beaubien-Souligny, Yoan Lamarche, André Y. Denault

**Affiliations:** a Université de Montréal, Montreal, Canada; b Division of Surgical Critical Care, Integrated Hospital Care Institute, Cleveland Clinic, Cleveland, United States of America; c Department of Anesthesiology and Intensive Care Medicine Division, Institut Universitaire de Cardiologie et de Pneumologie de Québec, Quebec City, Canada; d Division of Intensive Care Medicine, Centre Hospitalier Universitaire de Québec-Université Laval, Quebec City, Canada; e Division of Nephrology, Centre Hospitalier de l’Université de Montréal, Montreal, Canada; f Department of Surgery and Critical Care, Montreal Heart Institute, Montreal, Canada; g Department of Anesthesiology, Montreal Heart Institute, Montreal, Canada

**Keywords:** Venous congestion, Critical illness, Point-Of-Care ultrasound, Venous excess ultrasound, Lung ultrasound

## Abstract

Venous congestion is an important pathophysiological mechanism impacting organ function in critically ill patients. However, congestion physiology is complex, can affect multiple organ systems, and is subject to change rapidly over time. Contemporary multimodal assessment integrates both traditional approaches and emerging technologies to refine our understanding and improve clinical evaluation of this dynamic process. In this review, we explore how combining various ultrasound assessments can enhance the evaluation of congestion in critically ill patients. Topics include diastolic function evaluation with echocardiography, the Venous Excess Ultrasound (VExUS) assessment, and lung ultrasound.

## Background

Venous congestion in critically ill patients is a heterogeneous and multifaceted phenomenon that can manifest in distinct clinical forms [1]. Although it remains an active research topic, available evidence suggests that all organ systems are vulnerable to congestive organ injury [2]. To improve the evaluation of venous congestion in critically ill patients, attention has increasingly turned to multimodal assessment strategies. These approaches integrate traditional and emerging technologies to characterize the complex physiology of venous congestion in real time across multiple organ systems.

The objective of this expert opinion-based narrative review is to present how combining complementary ultrasound assessment approaches may enable a more comprehensive evaluation of congestion in critically ill patients. We discuss established and emerging tools – including echocardiographic diastology, lung ultrasound, and systemic veins ultrasound. We also present clinical algorithms and examples to illustrate how combining these assessments can potentially improve bedside evaluation of venous congestion in the intensive care unit (ICU).

## Definition of venous congestion

No universally standardized or consensus definition of venous congestion exists in the medical literature. That said, venous congestion is a term used to describe a pathophysiological state where hydrostatic pressure is increased in the veins and capillaries which in turn impact organ function [1]. Organ congestion can cause pulmonary edema, delirium, acute kidney injury, intestinal edema, hepatic dysfunction, impaired wound healing, and more [3]. Collectively, these clinical consequences can have serious implications for patient outcomes [2].

The pathophysiology of organ function impairment from venous congestion is thought to result from the interplay of several concurrent mechanisms. Increased venous pressure reduces the arteriovenous pressure gradient across vital organs, which may compromise adequate perfusion [4]. Importantly, true organ perfusion pressure is determined by the gradient between precapillary arteriolar pressure and postcapillary venular pressure rather than by the difference between mean arterial pressure (MAP) and central venous pressure (CVP). Because precapillary arteriolar pressures typically range from 35 to 40 mmHg, this arteriovenous gradient is substantially narrower than commonly assumed, which underlies the sensitivity of organ perfusion to elevations in venous pressure [5]. Sustained increases in capillary hydrostatic pressure can also lead to the development of interstitial edema in the setting of a dysfunctional endothelial barrier [6]. In encapsulated organs such as the kidney and the brain, interstitial edema can rapidly raise interstitial pressure, thereby exacerbating reductions in organ blood flow [7]. In addition, interstitial edema may impair tissue oxygenation by increasing diffusion distances within the interstitium [8].

Venous congestion has been extensively studied in cardiology populations. However, ICU patients are more heterogeneous, and congestion in this setting is often more complex, with varying clinical significance across different ICU subgroups. In this context, Guinot et al. applied machine learning to identify subtypes of congestion profiles in critically ill patients [1]. Characterizing these congestion endotypes may represent an important step toward a better understanding of congestion significance in ICU populations, and further studies are needed to validate and build upon these findings.

Correlation exists between cardiac filling pressures and venous congestion [9,10]; however, this relationship is not absolute. When cardiac function is preserved, excessive volume expansion can increase mean systemic filling pressure (MSFP) and capillary hydrostatic pressure, potentially causing congestive organ injury, even if CVP remains within the normal range [11]. Consistent with this, Guinot et al. identified a congestion endotype characterized by elevated portal pulsatility despite low to intermediate cardiac filling pressures, which was associated with a high incidence of acute kidney injury (AKI) [1]. Conversely, elevated cardiac filling pressures do not necessarily inform on organ-level repercussions. This may vary from one patient to another, which may depend in part on the chronicity of the condition. This highlights the theoretical advantage of assessing congestion through organ-level indices rather than relying solely on cardiac filling pressures [12].

More recently, some authors have introduced the concept of fluid tolerance [13]. Traditionally, fluid therapy has been guided primarily based on fluid responsiveness. However, it has been suggested that assessment should also consider whether a patient is likely to tolerate additional fluids. In other words, fluid tolerance aims to identify individuals for whom additional fluid administration would confer an increased risk of congestive organ injury. Accordingly, fluid responsiveness and fluid tolerance should be considered together to guide optimal management decisions.

Although they represent different concepts, the relationship between fluid responsiveness and fluid tolerance remains an area of active research. For example, recent evidence has shown that fluid loading is more likely to worsen venous congestion in non–fluid responders than in fluid responders in ICU patients [10]. Additionally, in a cohort of ICU patients, signs of venous congestion were significantly prevalent among both fluid responsive and fluid unresponsive patients [14]. This highlights the important concept that patients may demonstrate venous congestion despite being fluid responsive.

## Systemic veins ultrasound and the VExUS grading system

### Inferior vena cava

**Fig. 1:**
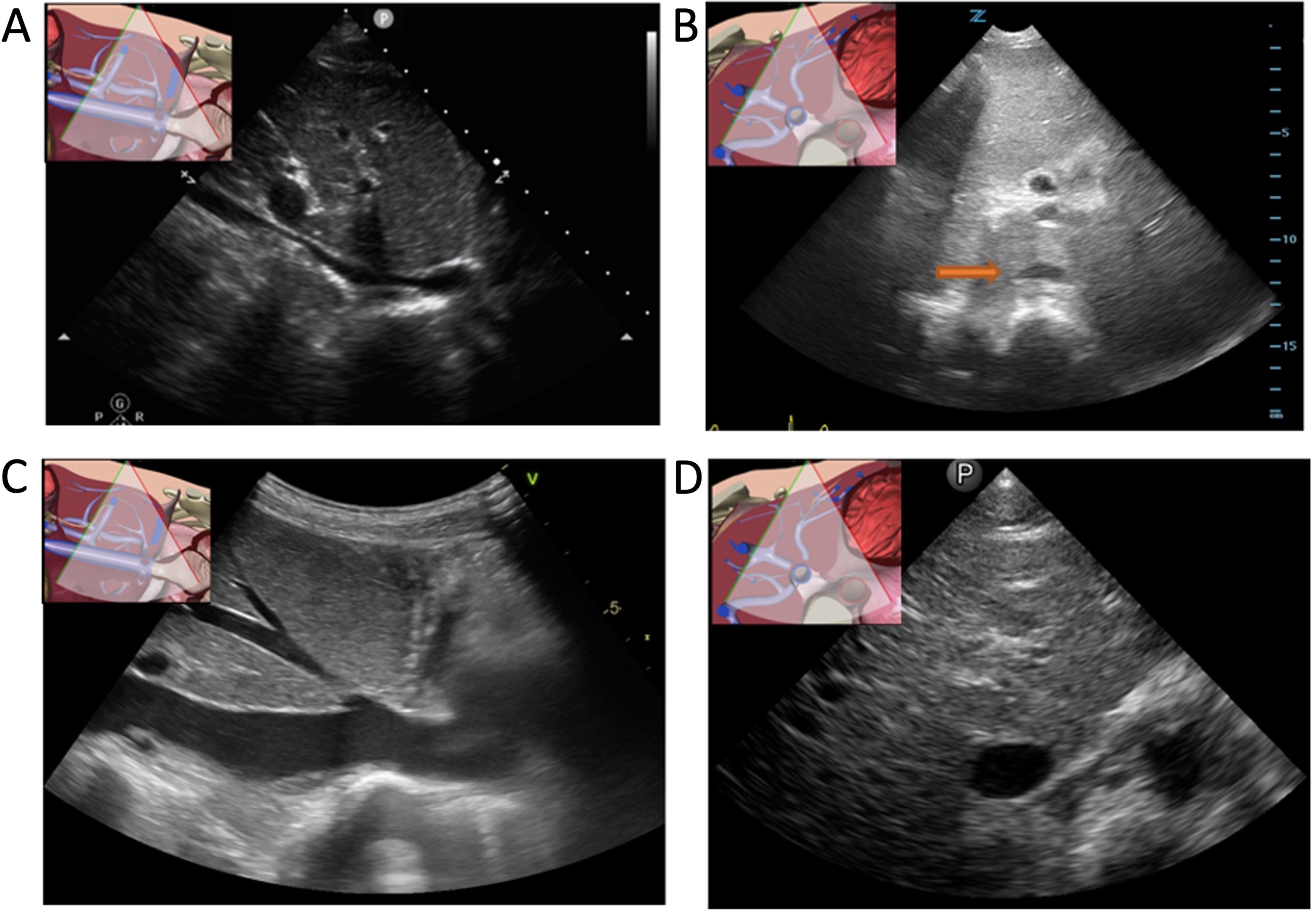
Inferior vena cava (IVC) assessment. (A) IVC demonstrating a maximal diameter inferior to 2.1 cm and (B) an oval appearance in transverse view is suggestive of normal or low RAP. (C) IVC distended above 2.1 cm and (D) with a round appearance in transverse view is suggestive of high RAP. Anatomic representations obtained using the Vimedix simulator (Elevate Healthcare). IVC, inferior vena cava; RAP, right atrial pressure.

Ultrasound evaluation of the inferior vena cava (IVC) is a relatively simple technique. Echocardiography guidelines [15] recommend estimating right atrial pressure (RAP) using a combined assessment of IVC diameter and respiratory variation, applicable to patients breathing spontaneously without any form of positive pressure respiratory support (Fig. 1). In contrast, in patients receiving positive pressure ventilation, IVC respiratory variation cannot be used to reliably estimate RAP [15]. In these patients, alternative approaches - ultrasound-based or otherwise - are required to assess right-sided filling pressures.

One important technical consideration in IVC ultrasonography is that the IVC is an oval-shaped cylinder, and measuring it off-axis can easily under- or overestimate its diameter. Experienced ultrasonographers often suggest also obtaining a transverse view to assess the shape of the IVC to avoid these issues (Fig. 1) [16].

### Principle of venous Doppler assessment

**Fig. 2:**
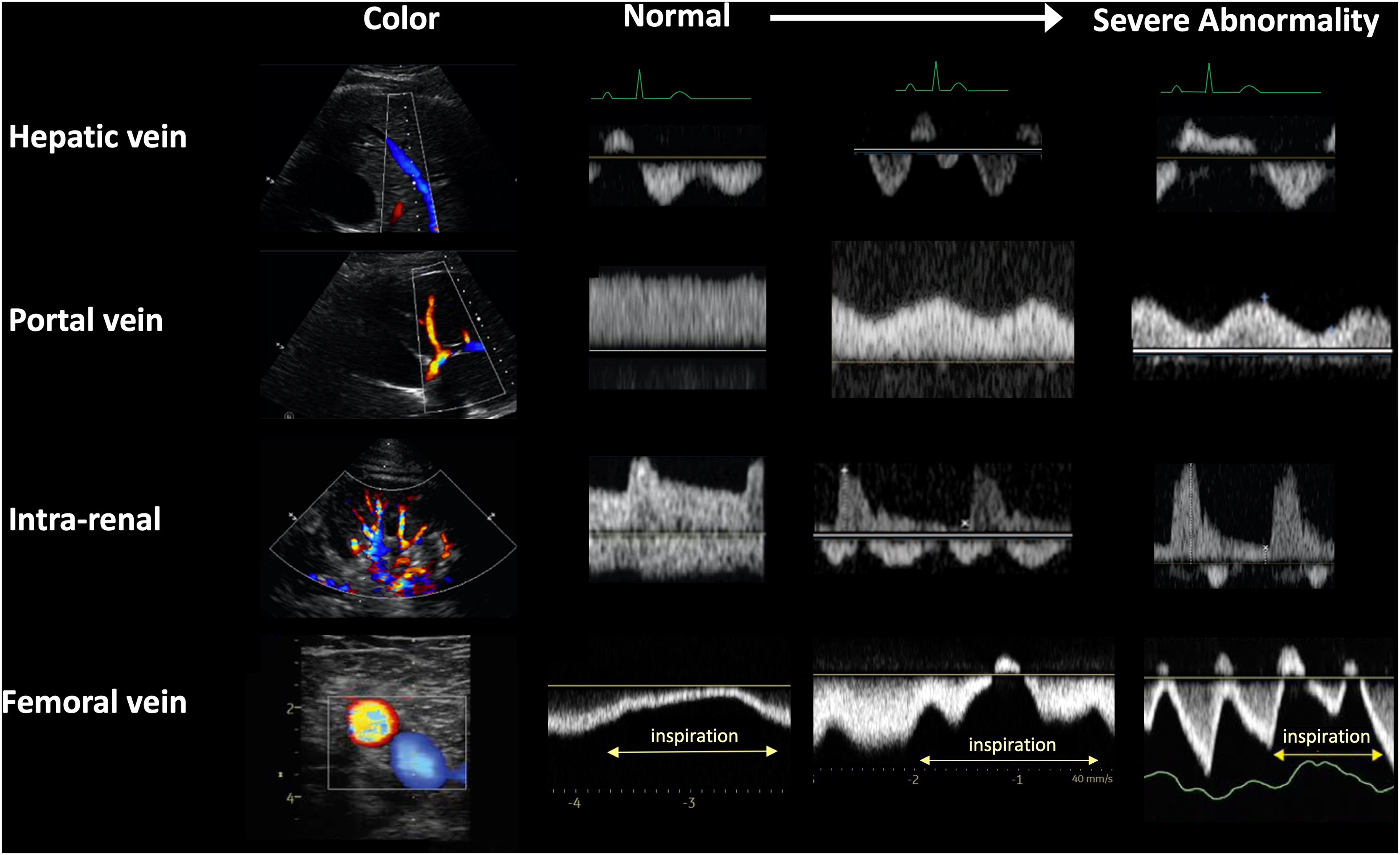
Doppler assessment of systemic veins with point-of-care ultrasound. Venous congestion causes abnormal waveform patterns.

In individuals with low RAP and normal venous compliance, large veins dampen RAP variations during the cardiac cycle. Consequently, RAP waveforms are normally not transmitted distally in the venous system [17]. In the late 1990s, abnormal venous Doppler patterns in the hepatic, portal, and common femoral veins were reported in patients with heart failure [18,19], and were later found to also occur in the interlobular veins of the kidney (Fig. 2) [20]. These alterations were classically described as arising from two interconnected phenomena. First, as right-sided filling pressures increase, atrial filling is impaired during systole due to the loss of the RA reservoir function. As a result, venous return predominantly shifts to diastole. Second, in patients with elevated systemic venous pressure normal venous compliance is lost. This leads to the transmission of abnormal RAP waveforms distally, affecting the hepatic, portal, intrarenal, and femoral veins [21].

### Hepatic vein Doppler

**Fig. 3:**
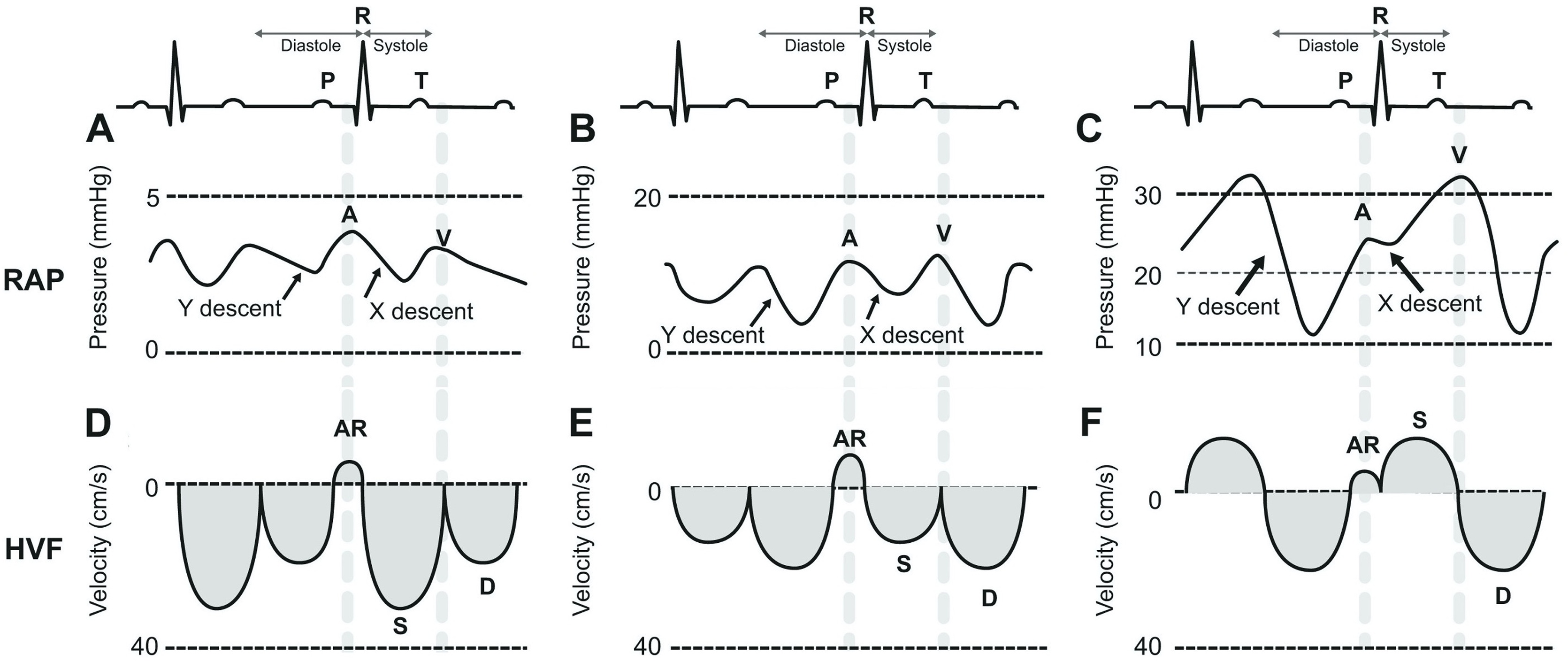
Right atrial pressure waveform and hepatic vein Doppler pattern. Correlation between right atrial pressure (RAP) waveform and hepatic venous flow (HVF) pattern. Typical patterns observed in normal patients (left panels: A, D), and in patients with mild (center panels: B, E) and severe (right panels: C, F) right ventricular dysfunction. AR, atrial reversal Doppler flow velocity; D, diastolic Doppler flow velocity; S, systolic Doppler flow velocity. Adapted from Couture et al. (Couture EJ, Gronlykke L, Denault AY. New developments in the understanding of right ventricular function in acute care. Curr Opin Crit Care 2022;28:331-9.)

The hepatic vein Doppler mirrors RAP changes during the cardiac cycle, as it is close to the heart and no valves are present (Fig. 3). In the normal patient, the first phase corresponds to atrial contraction, where retrograde flow is reflected by a small retrograde wave called the AR wave on hepatic vein Doppler. During ventricular systole, the X descent on the RAP tracing corresponds to the systolic antegrade flow called the S wave on the hepatic vein tracing. At the end of systole, the V wave on the RAP tracing is usually manifested by a short interruption of flow in the hepatic veins. Finally, upon tricuspid valve opening, the Y descent on RAP tracing is manifested by a diastolic antegrade flow called the D wave in the hepatic veins [17]. It is recommended to use an electrocardiogram to accurately identify the components of the hepatic Doppler profile, as the transition period from the S wave to the D wave can easily be mistaken for an AR wave [22,23]. When right-sided filling pressures increase, the hepatic vein profile continues to reflect changes in the RAP waveform. As venous return becomes dominant in diastole, the amplitude of the S wave decreases, with severe Doppler abnormality characterized by a reversed systolic phase [24].

### Portal vein Doppler

The portal vein is unique in that it is isolated from both the arterial and venous systems by the splanchnic capillaries and hepatic sinusoids, respectively. Portal vein flow is normally uninterrupted throughout the cardiac cycle and is either non-pulsatile or only minimally pulsatile [18,25,26]. Abnormal portal vein Doppler is defined as a pulsatile waveform with significant variations of velocities during the cardiac cycle with a pulsatility fraction (relative difference between peak and nadir over the peak) greater than 50% [25,27,28].

## Intrarenal vein Doppler

Under normal conditions, the intrarenal veins show continuous flow with little to no pulsatility. As a result of their proximity to the intrarenal arteries, the arterial waveforms are most often visible on the same pulse-wave Doppler tracing [25,29]. When abnormal Doppler profile develops, the intrarenal veins become pulsatile with periods of interrupted venous flow. Initially, flow interruptions are brief with venous flow still present during both systole and diastole. Severe Doppler abnormality corresponds to long interruptions with the venous signal being only present during diastole [17,25].

### Common femoral vein ultrasound

Ultrasound of the common femoral vein (CFV) offers a practical advantage over many other venous sites, as it is exceedingly simple and quick to perform [30]. Under normal conditions, the CFV Doppler waveform demonstrates continuous flow with only respirophasic variations in velocity. An abnormal CFV Doppler waveform is characterized by pulsatility, typically defined by the presence of cardiophasic retrograde flow at the end of expiration [19,[31], [32], [33]]. Other parameters, less frequently studied, have also been described for CFV ultrasound in the assessment of congestion [34], including CFV diameter [[35], [36], [37]] and femoral venous stasis index [38].

## Internal jugular vein ultrasound

Another venous ultrasound site is the internal jugular vein (IJV), which has been studied as a noninvasive method for estimating RAP in acutely ill patients [39]. Several measurements have been evaluated, including IJV height [39,40], static diameter [41], respiratory variation [42], and collapsibility during a sniff test [42]. Although promising, IJV ultrasound still requires further validation in critically ill patients, as techniques vary widely across studies and most evidence derives from small observational cohorts.

## Combining multiple sites of venous assessment: the VExUS grading system

The evaluation of venous congestion using ultrasound is enhanced when multiple venous Doppler sites are assessed in combination as opposed to interpreting them in isolation. This approach underpins the development of the Venous Excess Ultrasound (VExUS) grading system, which integrates ultrasound findings from the inferior vena cava, hepatic vein, portal vein, and intrarenal veins [43].

Cohort studies in cardiac surgery patients have reported associations between VExUS, or its individual components, and adverse outcomes such as acute kidney injury, duration of organ dysfunction, and postoperative complications [25,[43], [44], [45]].

However, studies evaluating VExUS in other critically ill populations—such as patients with severe acute kidney injury [46], sepsis [47,48], and mixed ICU cohorts [49,50]—have reported inconsistent associations with clinical outcomes.

In light of the evolving understanding of VExUS over recent years, we believe that several considerations warrant attention when interpreting the existing literature and integrating VExUS into clinical practice.

## Revisiting the conceptualization of VExUS grades: Likelihood of congestion rather than a severity spectrum

Since the original publication of the VExUS score in 2020, understanding of this tool has continued to evolve [43]. In its initial description, VExUS grades were presented as a severity spectrum of venous congestion, ranging from grade 0 (no congestion) and grade 1 (at risk) to grade 2 (moderate congestion) and grade 3 (severe congestion).

We now hypothesize that it is more appropriate to conceptualize VExUS grades as reflecting the *likelihood* of clinically meaningful venous congestion rather than a linear severity continuum. Under this framework, grades would be interpreted as follows: grade 0, no congestion; grade 1, at risk; grade 2, possible congestion; and grade 3, probable congestion.

The rationale for this distinction lies primarily in the high risk of false-positive classification associated with VExUS grade 2. This grade, defined by the presence of a single severely abnormal venous Doppler pattern, is particularly vulnerable to misclassification. Hepatic vein, portal vein, and intrarenal venous Doppler patterns may each be influenced by confounding factors unrelated to venous congestion, as described in detail in prior work [17]. In this context, characterizing these patients as having possible rather than moderate congestion more accurately reflects the underlying diagnostic uncertainty.

This conceptual distinction has important implications for both clinical and research interpretation. In ICU cohorts where venous congestion is uncommon, the proportion of patients with VExUS grade 3 may be very small. In such settings, outcome analyses often rely on a composite threshold of VExUS grade ≥2. However, this strategy carries a risk of introducing a substantial number of false-positive classifications, particularly when abnormal VExUS scores are largely composed of grade 2 and include only a small number of grade 3. Reframing VExUS grade 2 as possible congestion may therefore enhance the interpretability of study findings and reduce misclassification in outcome analyses.

In spite of this, it should be emphasized that this framework remains hypothetical and requires further validation. Notably, alternative approaches may also be used to enhance the validity of the VExUS grading system. For example, Si et al. applied a point-based method to define VExUS assessment in a recently published cohort study [10].

## Statistical power and prevalence of abnormal venous Doppler patterns

**Fig. 4:**
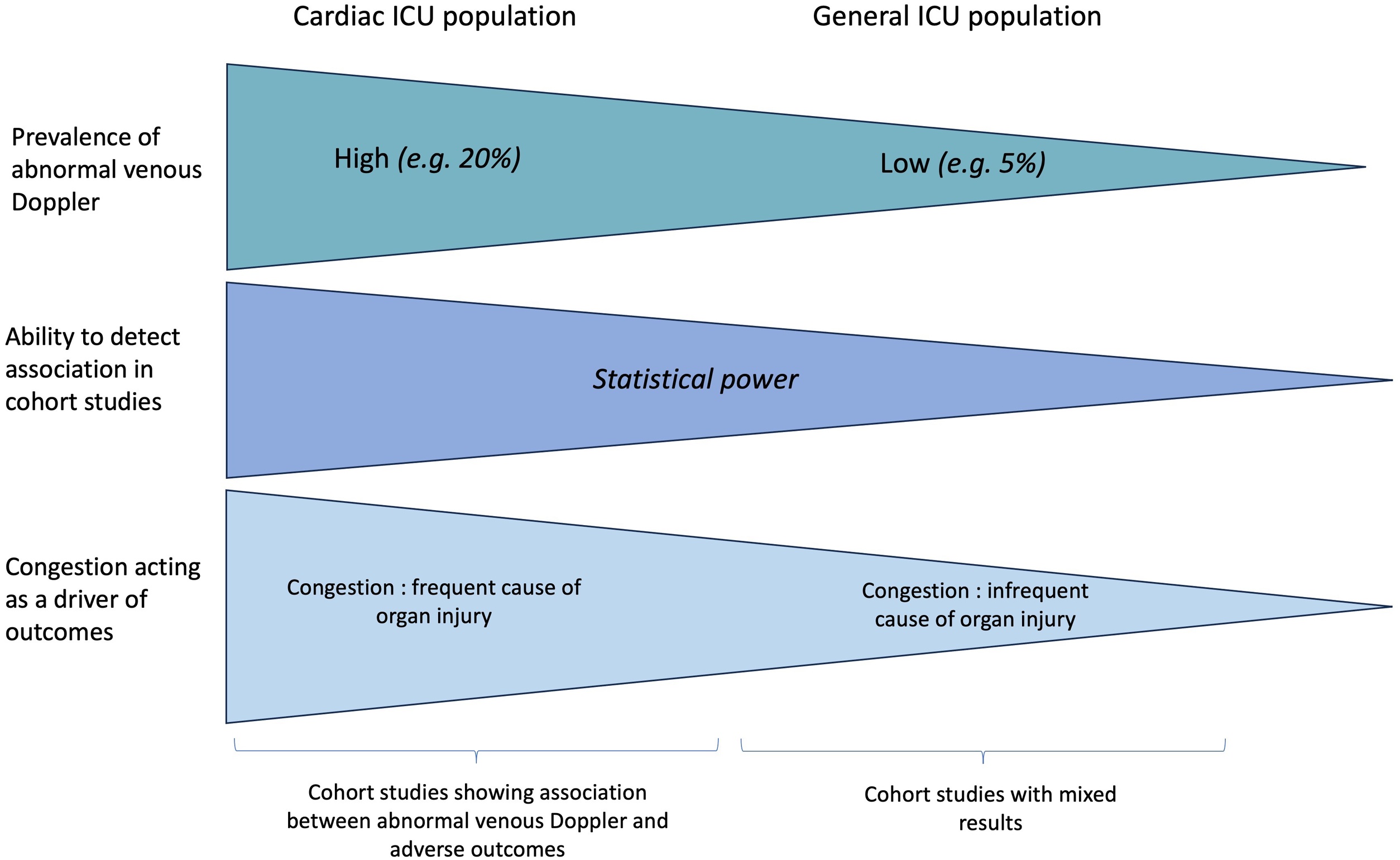
Venous Doppler abnormalities and association with adverse outcomes in different ICU populations. Cohort studies on venous Doppler yield different results depending on the ICU subgroup under study. Possible factors underlying these results are illustrated here. ICU, intensive care unit.

An additional consideration relates to the prevalence of abnormal venous Doppler findings in the populations under study. In several investigations of VExUS in non-cardiac ICU cohorts, abnormal VExUS scores – particularly VExUS grade 3 – were infrequent, and no association with adverse outcomes was identified [48,49]. Low exposure prevalence substantially limits statistical power, increases estimate imprecision, and reduces the ability to detect clinically meaningful associations, even when such relationships truly exist. Consequently, negative findings in these ICU subgroups should be interpreted with caution, as they could reflect insufficient power rather than the absence of a true association between abnormal VExUS and clinical outcomes (Fig. 4).

## Context-dependent pathophysiological relevance of venous congestion

Beyond issues of statistical power, the pathophysiological relevance of venous congestion is likely to be context dependent. The strength of the association between VExUS abnormalities and outcomes depends on the extent to which venous congestion represents a dominant pathophysiological driver of adverse outcomes within the ICU subgroup under study. In non-cardiac ICU setting, venous congestion may represent a secondary contributor of organ injury, thereby attenuating the observable association between VExUS and clinical outcomes. In other words, the same clinical outcome can arise from different causal pathways in different ICU populations. This does not necessarily invalidate the VExUS – it defines its contextual relevance (Fig. 4).

## Suggested approach to patients presenting abnormal VExUS

As the VExUS score has gained wider adoption, some clinicians have interpreted it primarily as a tool for assessing volume status; however, this interpretation is an oversimplification. As pointed out in a recent perspective article by Guinot, ''VExUS reflects the interaction of venous return and cardiac function rather than simple volume overload parameter'' [51]. Indeed, it is important to recognize that multiple mechanisms can contribute to systemic venous congestion and should be taken into account when interpreting abnormal VExUS findings [5,11,51].

**Fig. 5:**
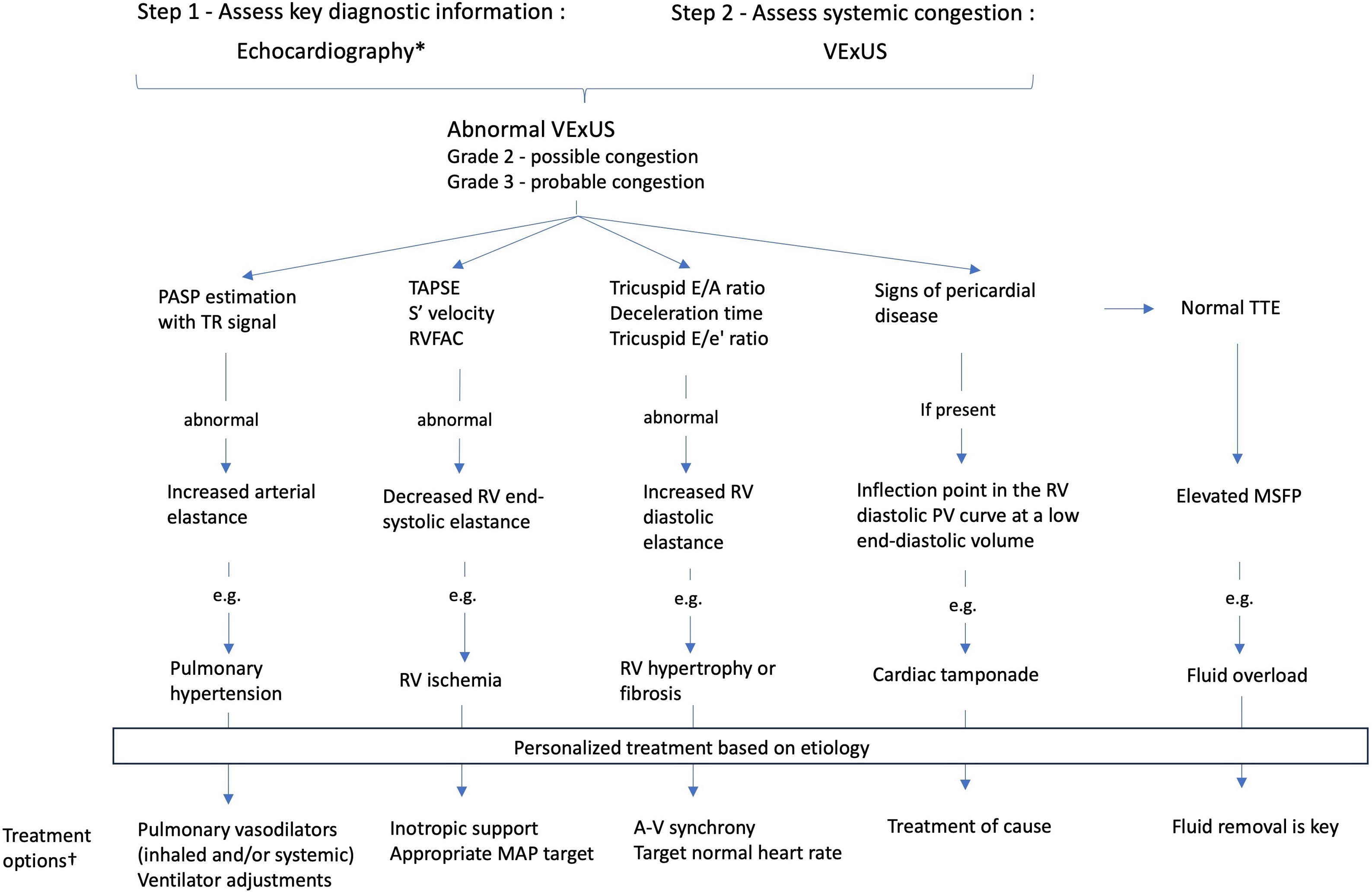
Proposed expert opinion–based approach for integrating echocardiography and VExUS assessment. *Other test/investigations may be essential according to context. †Significant overlap between treatment strategies may be used. Lists presented here are not exhaustive. To be personalized according to clinical context. Mechanical support options are not discussed here. Disclaimer: This algorithm is based on expert opinion, and prospective validation is warranted. PASP, pulmonary artery systolic pressure; TR, tricuspid regurgitation; TAPSE, tricuspid annular plane systolic excursion; RVFAC, right ventricular fractional area change; TTE, transthoracic echocardiogram; MSFP, mean systemic filling pressure; PV, pressure-volume; RV, right ventricular; MAP, mean arterial pressure; A-V atrioventricular.

We developed an algorithm that combines echocardiography and VExUS and serves as a conceptual model, which we hypothesize may aid in understanding and evaluating patients with abnormal VExUS scores (Fig. 5). This algorithm proposes additional echocardiographic markers to consider in patients with abnormal VExUS, corresponding to different pathophysiologic pathways potentially involved in systemic venous congestion. It also suggests treatment strategies tailored to the mechanisms involved. The algorithm highlights the potential role of right ventricular systolic and diastolic elastance, as well as pulmonary arterial elastance, in systemic venous congestion [52]. Awareness of pericardial disease remains a critical caveat. In some cases, pure elevation of mean systemic filling pressure (MSFP) may drive congestion [11]. Significant overlap may be present between congestion mechanisms and treatment strategies. Information on the echocardiographic measurements referenced in the algorithm can be found in current guidelines [15,53]. More details about the role of echocardiography in this context is discussed further in the following sections.

## Lung ultrasound

Lung congestion represents a substantial burden among ICU patients. Evidence from transpulmonary thermodilution studies indicates that extravascular lung water is a predictor of mortality across diverse populations of critically ill patients [54,55].

**Fig. 6:**
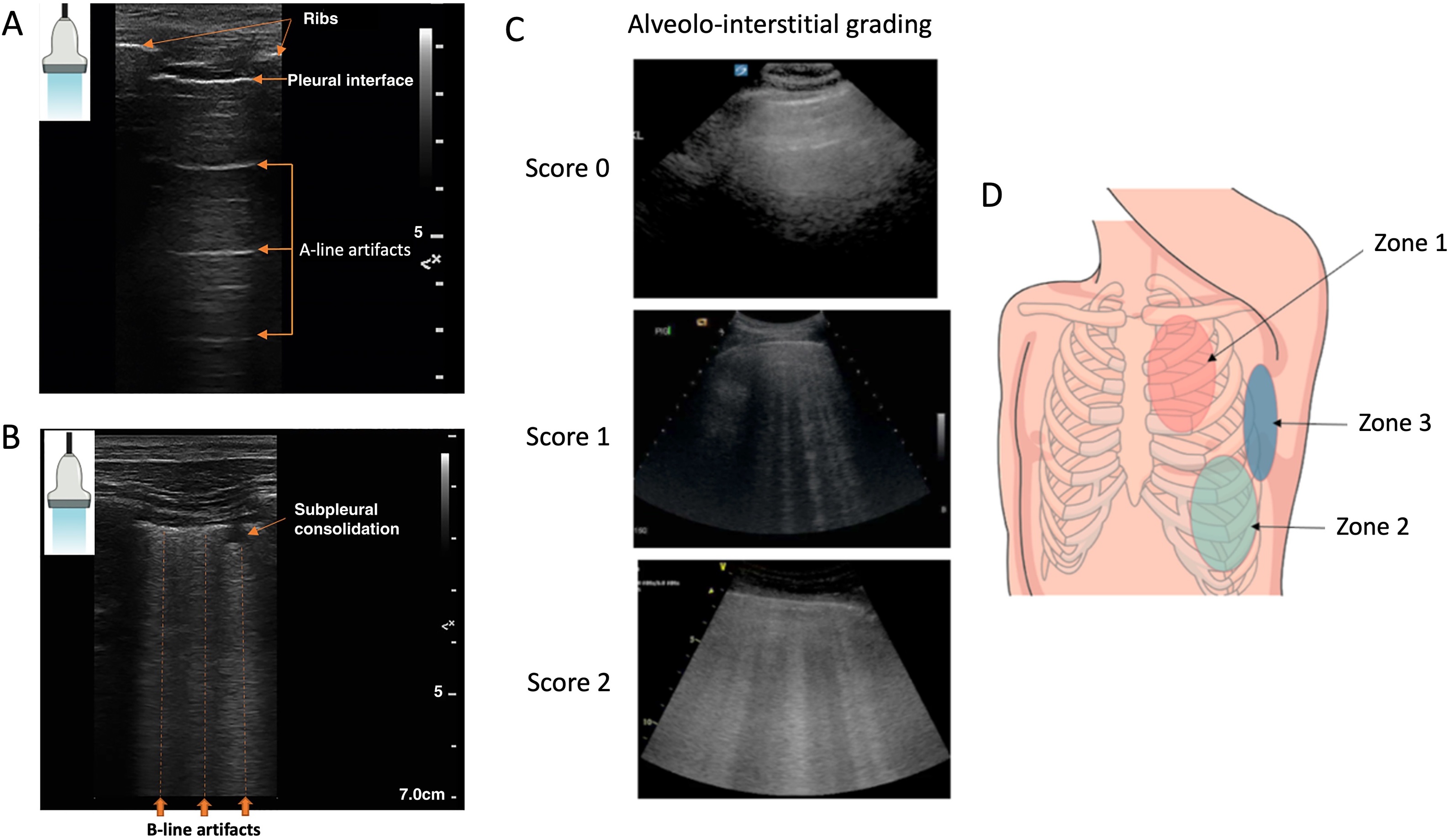
Lung ultrasound for evaluation of congestion. (A) Normal appearance including rib shadows, pleural interface, and horizontal a-line artifacts. (B) Presence of alveolar-interstitial syndrome associated with the appearance of at least 3 vertical b-line artifacts that arise from the pleura. (C) Lung ultrasound scoring system to quantify loss of lung aeration (Score 0 if A-lines or ≤ 2 B-lines; Score 1 if ≥ 3 distinct B-lines; Score 2 if presence of coalescent B-lines). (D) Three per side scanning protocol.

Lung ultrasound (LUS) is one of the most readily accessible lung imaging modalities in the intensive care unit [56]. When normal lung aeration is impaired, the resulting acoustic impedance mismatch generates ultrasound artifacts called B-lines [57]. B-line artifacts can result from various processes such as pulmonary edema, interstitial lung disease, pneumonia, acute respiratory distress syndrome (ARDS) and others [58]. Multiple lung ultrasound scanning protocols have been proposed. A simplified approach using three zones per side has demonstrated diagnostic accuracy comparable to that of protocols employing a greater number of scanning zones for the assessment of lung congestion [59]. For each zone assessed, a commonly used lung ultrasound scoring system is applied to quantify loss of lung aeration. Scores are assigned as follows: normal aeration with A-lines or two or fewer B-lines (score 0); moderate loss of aeration with three or more well-spaced B-lines (score 1); and severe loss of aeration with coalescent B-lines (score 2) [58] (Fig. 6). A score of ≥1 in multiple lung zones has been shown to correlate with invasively measured extravascular lung water in general ICU population [[60], [61], [62]].

As noted above, when using LUS to assess lung congestion, it is crucial to recognize that the presence of bilateral B-lines across multiple lung zones can result from various conditions. Clinical and paraclinical evaluation is therefore essential for the clinician to determine whether B-lines may be explained by an alternative diagnosis. Certain ultrasound features can provide additional clues to differentiate the underlying etiology. For instance, subpleural consolidations, patchy patterns with spared areas, or irregular and thickened pleura may suggest conditions such as interstitial lung disease (ILD), ARDS, or pneumonia [63,64]. LUS also enables sensitive detection of pleural effusions with accuracy comparable to thoracic computed tomography (CT), offering further insight into the underlying cause [65]. When confounding conditions are suspected, echocardiographic assessment of left ventricular (LV) diastolic function and estimation of left atrial pressure (LAP) can serve as an additional bedside tool, as further discussed in the next section.

**Fig. 7:**
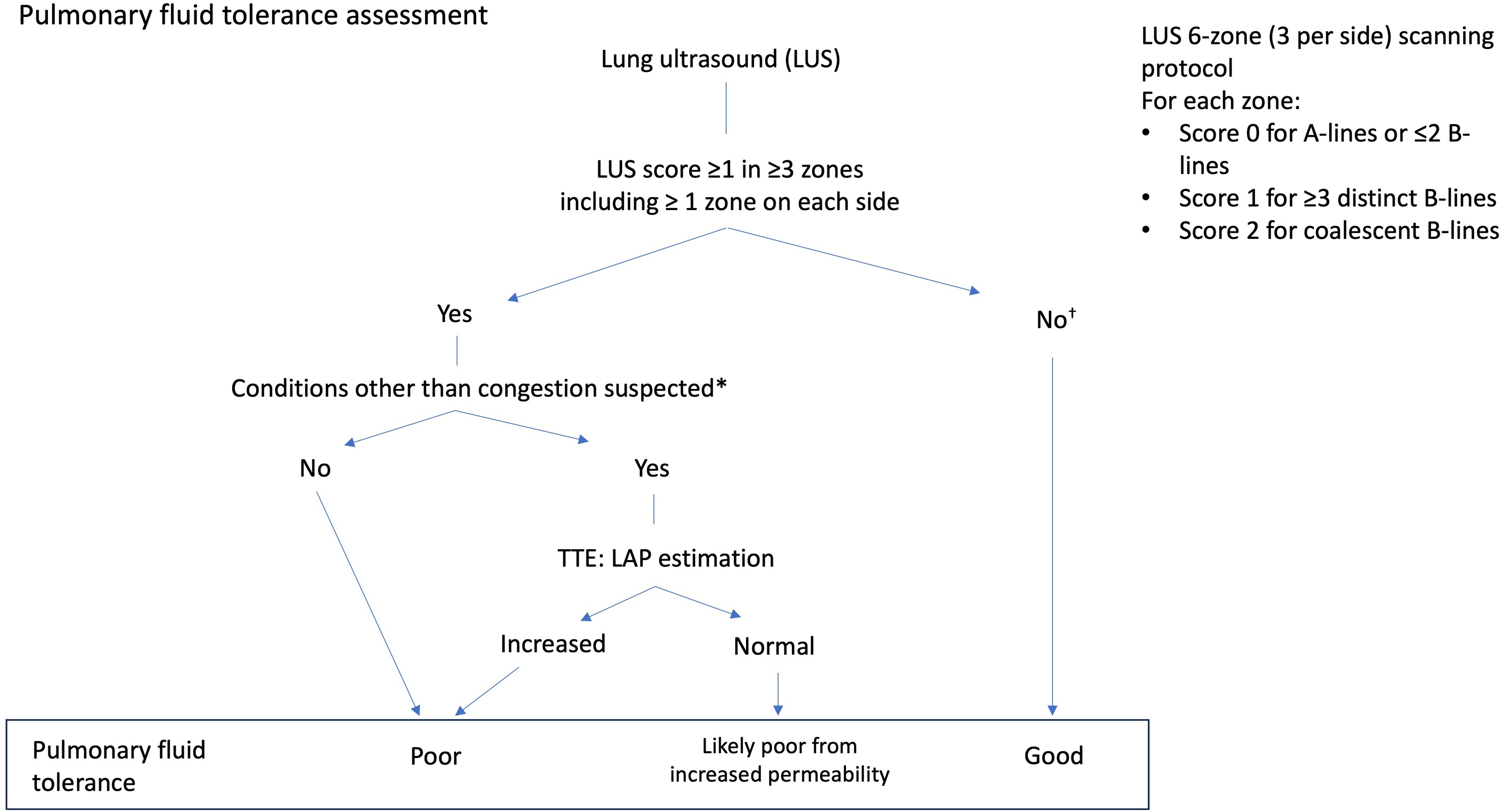
Algorithm for assessing pulmonary fluid tolerance using lung ultrasound. *Examples include pneumonia, ARDS, and ILD. †Pitfall: Regardless of LUS findings, a patient with elevated LAP would be considered to have poor pulmonary fluid tolerance, suggesting that TTE may also be valuable in that situation. Disclaimer: This algorithm is based on expert opinion, and prospective validation is warranted. ARDS, acute respiratory distress syndrome; ILD, interstitial lung disease; LAP, left atrial pressure; TTE, transthoracic echocardiogram.

Building on the previously described concept of fluid tolerance, pulmonary fluid tolerance refers to the lungs’ ability to accommodate additional volume expansion without developing congestion. This tolerance is influenced not only by increases in hydrostatic pressure but also by changes in microvascular permeability. Disruption of normal capillary permeability occurs in conditions such as ARDS, pneumonia, and other inflammatory states. Consequently, pulmonary fluid tolerance may be poor in these settings even with normal LV filling pressures, depending on the degree of microvascular permeability alteration [10,54]. We developed an algorithm that integrates the key principles for interpreting pulmonary fluid tolerance using LUS (Fig. 7). We applied this algorithm in an interventional trial to guide clinicians in assessing fluid tolerance in patients admitted to the hospital with AKI (NCT06411080).

## Echocardiographic assessment of LV diastolic function and estimation of left atrial pressure

As part of comprehensive assessment for lung congestion, the echocardiographic assessment of LV diastolic function and estimation of LAP is a valuable addition to ICU patient assessment [66,67]. Echocardiographic assessment of LV diastolic function is a multiparametric approach that integrates two-dimensional imaging and Doppler measurements and has been increasingly applied in critical care settings.

Most diastology algorithmic approaches were developed and validated in non-critically ill patients. The most recent American Society of Echocardiography (ASE) guidelines on diastolic function, published in 2025, update the previous ASE guidelines from 2016 and 2009 [[68], [69], [70]]. The stepwise diagnostic approach includes indices of LV relaxation (e′), variable describing the pattern of LV diastolic filling (mitral E/A ratio), and indices reflecting elevated LV filling pressures and left atrial pressure (E/e′ ratio, left atrial reservoir strain [LARS], indexed left atrial volume [LAVi], and LV mass index). A separate algorithm is used to determine gradation of severity and LAP estimation, additionally incorporating tricuspid regurgitation (TR) peak velocity or estimated pulmonary artery systolic pressure, pulmonary vein S/D ratio, and isovolumetric relaxation time (IVRT) [68].

LV diastology assessment has limitations in critically ill patients, as most variables are dynamically affected by rapidly varying preload, afterload, and contractility related to acute illness and treatments. Patient’s classification can radically change during their ICU course, with one study in septic shock patients showing an initial rate of diastolic dysfunction of 60% that fell to 40% by day 3 [71]. One challenge lies in distinguishing diastolic dysfunction, which reflects intrinsic myocardial abnormalities, from filling pressures, which are dynamic hemodynamic conditions influenced by loading status. These elements are intrinsically linked, and most markers overlap in their assessment; however, some general concepts are worth mentioning. In general, intracavitary or intravascular Doppler measurements, such as E/A, peak TR velocity, deceleration time (DT), and hepatic vein flow, are more preload- and afterload-sensitive. In ICU patients, peak TR velocity is frequently abnormal due to intrapulmonary vascular changes related to acute parenchymal disease or mechanical ventilation [[72], [73], [74]]. Markers that assess structural elements through 2D echocardiography or Tissue Doppler imaging (TDI), such as LAVi or e’, tend to better reflect underlying myocardial abnormalities and are less preload- and afterload-sensitive. Increased LAVi, in the absence of chronic atrial arrhythmia and mitral valve disease, can be a marker of chronically elevated LAP secondary to underlying myocardial pathology [75,76]. TDI e’ velocity is a myocardial velocity measurement that is highly indicative of impaired myocardial relaxation and less affected by dynamic preload and afterload changes. In any case, reliance on a single parameter should be avoided, as no individual measure is sufficiently accurate to provide a comprehensive assessment [68].

**Fig. 8:**
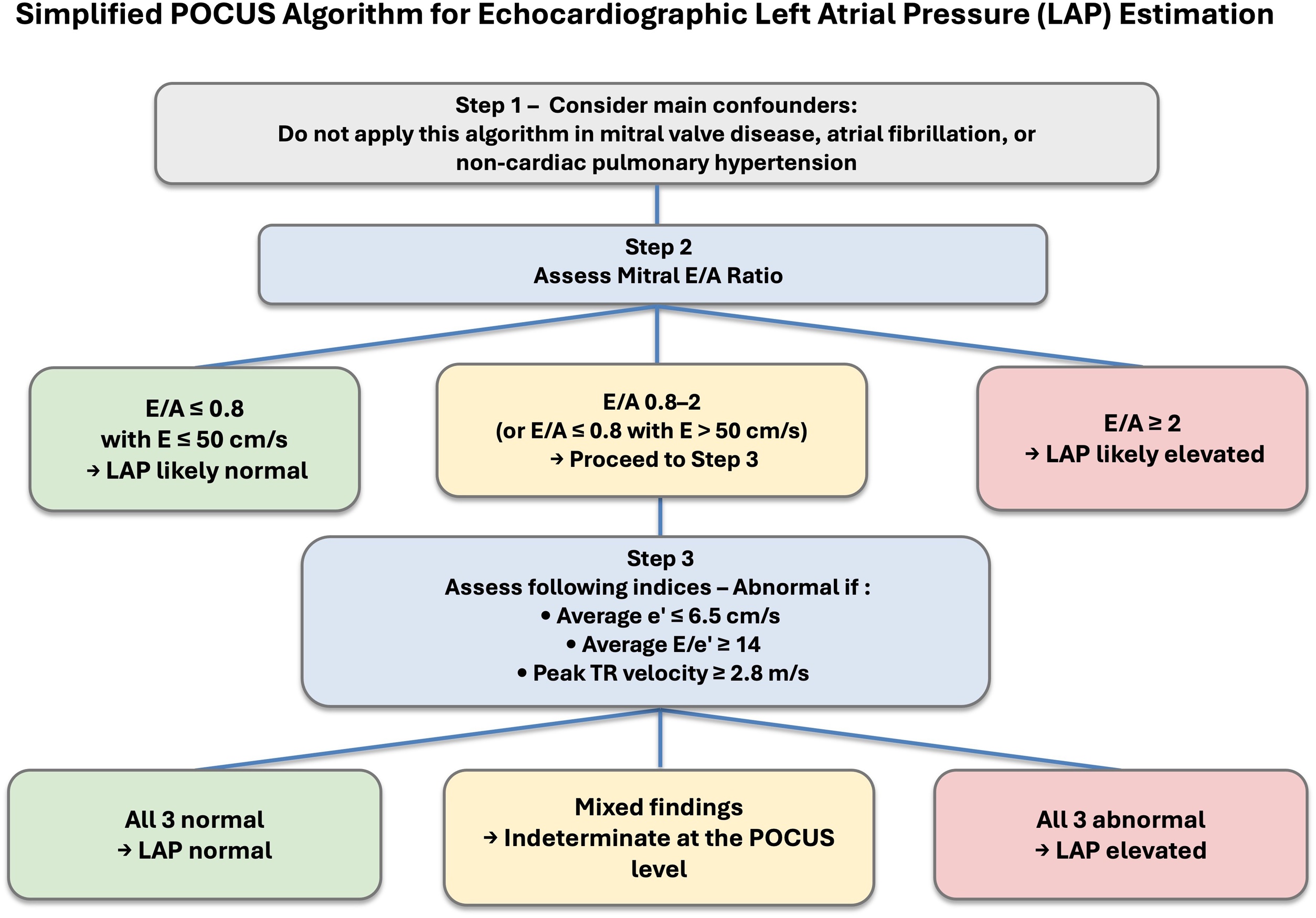
Simplified algorithm for echocardiographic estimation of left atrial pressure (LAP) designed for point-of-care ultrasound (POCUS). Disclaimer: This algorithm is based on expert opinion, and further validation in critically ill populations is warranted. LAP, left atrial pressure; POCUS, point-of-care ultrasound; TR, tricuspid regurgitation.

That said, in the setting of critical illness, the identification of elevated LV filling pressures may be more clinically relevant than the grading of diastolic dysfunction, particularly for guiding hemodynamic management. Among available echocardiographic indices, the E/e′ ratio appears to be a useful marker for assessing LV filling pressures in the ICU setting. However, it should be noted that using E/e' to estimate pulmonary artery occlusion pressure (PAOP) is characterized by a substantial grey zone, in which different thresholds involve a trade-off between sensitivity and specificity, and no single cutoff is universally applicable [77,78]. In addition to E/e′, other echocardiographic parameters have been evaluated for estimating PAOP in critically ill patients. For example, Brault et al. reported that the best echocardiographic predictors of normal PAOP were a lateral e′ velocity >8 cm/s in patients with preserved LV ejection fraction (≥45%) and an E/A ratio ≤1.5 in those with reduced LV ejection fraction (<45%) [79]. In light of these considerations, we propose a simplified expert opinion–based algorithm to guide POCUS assessment of LAP in critically ill patients (Fig. 8).

In ICU patients, considerable interest has focused on the association between left ventricular diastolic dysfunction and failure to wean from mechanical ventilation. This relationship is primarily driven by changes in left ventricular loading conditions that occur with the removal of positive pressure ventilation. Multiple mechanisms contribute, including increased LV preload, increased LV afterload, and enhanced sympathetic stimulation [80]. Collectively, these changes can precipitate lung congestion, thereby increasing the risk of weaning failure. A systematic review and meta-analysis by Sanfilippo et al. in 2021, which included 11 studies, evaluated the relationship between echocardiographic parameters and weaning outcomes. Among both systolic and diastolic variables, an elevated E/e′ ratio showed the strongest association with weaning failure, followed by reduced e′ values [81]. These results suggest that echocardiographic diastolic indices may help identify patients at higher risk of pulmonary congestion during liberation from mechanical ventilation, a common and clinically challenging scenario in the ICU.

## Right ventricular diastolic function assessment

Echocardiographic assessment of right ventricular (RV) diastolic function has been considerably less well studied than that of the left ventricle. Nonetheless, in patients presenting with signs of systemic venous congestion, we hypothesize that right-sided diastolic indices may provide complementary information as part of a comprehensive hemodynamic assessment, provided their limitations are acknowledged and interpreted within an integrated framework (Fig. 5).

Standard right ventricular diastolic evaluation includes pulsed-wave Doppler assessment of transtricuspid inflow, tissue Doppler imaging of the lateral tricuspid annulus, and pulsed-wave Doppler analysis of hepatic vein flow patterns, the latter also being assessed as part of the VExUS evaluation. Grading of right-sided diastolic dysfunction relies on the tricuspid E/A ratio and deceleration time, with a restrictive filling pattern characterized by a tricuspid E/A ratio > 2.1 and a deceleration time <120 ms. Criteria also include an elevated tricuspid E/e′ ratio (> 6) and diastolic flow predominance in the hepatic veins [15,24].

These parameters should be measured at end-expiration and averaged over at least five consecutive cardiac cycles. Hemodynamically significant tricuspid valve disease and atrial arrhythmias represent important confounders. Accordingly, it is recommended that assessment of RV diastolic function not rely on any single diastolic parameter in isolation [15].

Importantly, data supporting the validity of these right-sided diastolic markers in critically ill patients remain sparse. In a small observational study including 53 non-cardiac surgery ICU patients, Sade et al. demonstrated that a tricuspid E/e′ ≥ 4 predicted a right atrial pressure ≥10 mm Hg with a sensitivity of 88% and a specificity of 85%. In this study, tricuspid E/e’ had a strong correlation with RAP whether the patient was mechanically ventilated or not [82]. That being said, the validity of echocardiographic markers of RV diastolic function requires further validation in critically ill populations.

## Atrial strain analysis

One echocardiographic parameter mentioned in earlier sections is atrial strain analysis, for which a few clarifications are warranted. Of note, atrial strain analysis in critically ill patients remains an evolving area of research, and its accuracy and clinical applications still require further investigation.

In echocardiography, strain refers to a measure of myocardial deformation during the cardiac cycle. This deformation can be measured by echocardiography using speckle tracking technology [83,84].

Atrial strain analysis has been used as a surrogate marker for diastolic dysfunction. When elevated atrial pressures are required for adequate ventricular filling, increased atrial pressures lead to reduction in atrial compliance, which is reflected on atrial deformation patterns [85].

The morphology of the atrial strain curve reflects the three functional phases of the atrium: as a reservoir in systole, a conduit in early diastole, and a booster pump (atrial contraction) in late diastole [86]. Atrial reservoir strain reflects atrial relaxation and compliance, to which ventricular systolic function via annular descent also contributes. Atrial conduit strain is highly dependent on ventricular relaxation and chamber stiffness. Atrial contraction strain represents intrinsic atrial contractility but is also influenced by ventricular end-diastolic pressure and compliance [87]. In atrial strain analysis, two types of zero reference points are commonly used, depending on whether the strain calculation is initiated from the QRS complex or the P wave [88]. When the R wave is used as the starting point, all strain values appear as positive. In contrast, using the P wave as the reference produces an asymmetric sinusoidal strain curve. Independent of the chosen zero reference point, reservoir, conduit, and contractile strain can be considered interchangeable when interpreted as scalar values.

**Fig. 9:**
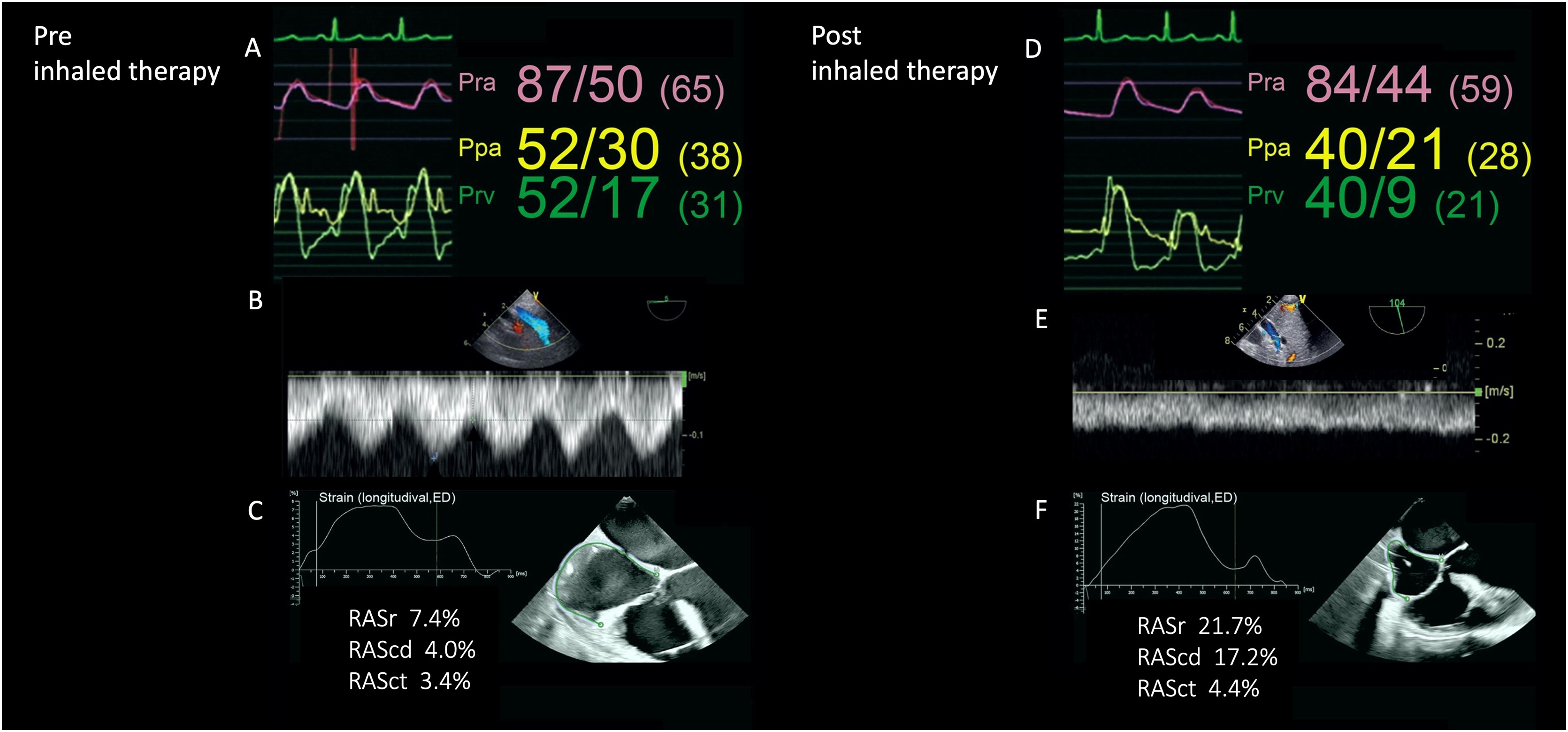
Multimodal assessment of venous congestion: Illustrative case example. This example is adapted from a real clinical case of a cardiac surgery patient evaluated before and after inhaled therapy with a combination of epoprostenol and milrinone. Pre-intervention, the patient demonstrated: (A) elevated pulmonary artery pressure (Ppa) with an abnormal right ventricular (RV) pressure waveform, (B) severe portal vein pulsatility exceeding 50%, and (C) markedly impaired right atrial strain, with a reservoir strain (RASr) of 7.4%. Post-intervention, the patient showed: (D) a reduction in Ppa and improvement in the RV pressure waveform, (E) normalization of portal vein flow, and (F) improved right atrial strain, with RASr increased to 21.7%. Ppa, pulmonary artery pressure; Pra, radial arterial pressure; Prv, right ventricular pressure; RAScd, right atrial conduit strain; RASct, right atrial contractile strain; RASr, right atrial reservoir strain. Adapted from Denault et al. Transesophageal Echocardiography Multimedia Manual, Third edition: A Perioperative Transdisciplinary Approach. London: Taylor & Francis; CRC Press; 2026.

Left atrial strain analysis circumvents some of the limitations of standard LV diastolic function assessment with echocardiography and could potentially represent an interesting complementary tool in ICU patients [69]. Data on right atrial strain analysis remain limited, although it may be useful for assessing RV diastolic function (Fig. 9).

## Conclusion

The body of literature on venous congestion is rapidly expanding, alongside the development of new technologies aimed at improving its understanding and assessment at the bedside. Further studies are needed to validate these emerging tools, to appreciate their limitations, and to clarify how multimodal assessment can inform preventive and therapeutic strategies with the ultimate goal of improving patient outcomes.

## Authors contributions

All authors contributed to the preparation of the original draft, as well as to the review and editing of the manuscript. All authors have read and approved the final version.

## Consent for publication

Informed consent was obtained from all patients whose ultrasound images or other data are presented in this manuscript.

## Ethics approval

Not applicable

## Funding

Dr. Denault's work is supported by the Montreal Heart Institute Foundation and Richard I. Kaufman Endowment Fund in Anesthesia and Critical Care.

Dr. Beaubien-Souligny’s work is supported by the Fonds de recherche du Québec – Santé (FRQS) (https://doi.org/10.69777/312266).

## Availability of data and material

Data used to support the findings of this study are available from the corresponding author upon request.

## Data availability

The data that has been used is confidential.

Data will be made available on request.

## Declaration of competing interest

Dr. Denault is a speaker and consultant for Elevate Healthcare and BD.

Dr. Beaubien-Souligny received honoraria from GSK (2022).

Dr. Couture is on the Cardiac Advisory Board of Edwards Lifesciences (2023).
